# Using the Solitary Pollinator *Osmia lignaria* to Bridge Agricultural Practice and Ecological Theory

**DOI:** 10.1002/ece3.74059

**Published:** 2026-07-25

**Authors:** Natalie Van Pelt, Magda Argueta‐Guzmán, Trizthan Jimenez Delgado, Lindsie McCabe, Marilia Palumbo Gaiarsa

**Affiliations:** ^1^ Department of Life and Environmental Sciences, School of Natural Sciences University of California Merced California USA; ^2^ USDA ARS Pollinating Insects Research Unit Logan Utah USA

**Keywords:** cavity‐nesting, developmental plasticity, environmental stressors, floral resource availability, foraging behavior, reproductive success

## Abstract

With global pollinator populations in decline and colony collapse disorder threatening honey bee colony loss, understanding the biology and management of alternative pollinators has become increasingly urgent. Although commercially managed species—particularly honeybees and a few bumblebee species—have been studied extensively, the vast majority of bee species (~80%) are solitary. As such, solitary bees provide a more appropriate foundation for informing conservation and management strategies than their social and eusocial counterparts. In particular, the native North American Blue Orchard Bee *Osmia lignaria* Say (Megachilidae) has received increasing attention as a viable pollinator for a wide range of crops. However, solitary bee species such as *Osmia* remain underutilized as model systems for linking behavioral ecology to pollination function, despite their growing use in agricultural systems. In this synthesis, we review current knowledge on *Osmia lignaria* 's developmental plasticity, nesting behavior, foraging strategies, pollination services, and major stressors. We place special emphasis on how environmental conditions and management practices influence its performance as a crop pollinator and as a model organism for ecological research. We also identify key knowledge gaps to help guide future ecological research and inform more sustainable pollination strategies in both agricultural and natural systems.

## Graphical abstract

We synthesize key ecological and life‐history aspects of the solitary bee *Osmia lignaria* , including early developmental stages, nesting biology, and foraging strategies. We further relate these traits to pollination services across agricultural and natural ecosystems and identify the most important current stressors affecting this species. Our work highlights this solitary bee species as a model system to link behavioral ecology to pollination function and applied agricultural practice.

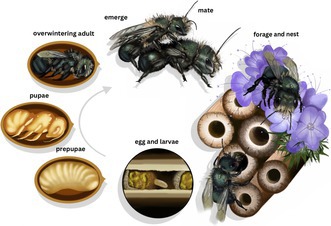

## 1 Introduction

Pollinators are essential to the functioning of terrestrial ecosystems and the productivity of many crops, with over 85% of flowering plants depending on animal pollination (Ollerton et al. 2011). Among pollinators, bees are one of the most effective and ecologically significant groups, facilitating plant reproduction in both wild and agricultural systems (Klein et al. 2007; Winfree et al. 2007). However, the continued loss of pollinator populations due to habitat degradation, climate change, disease, pesticide exposure, and nutritional deficits driven by habitat degradation and monoculture crops presents an ecological crisis with direct implications for food security (Potts et al. 2010; Janousek et al. 2023; Reilly et al. 2020). Addressing this crisis requires a deeper understanding not only of species‐level declines but also of the behavioral and physiological mechanisms that determine pollinator performance and persistence in dynamic environments. These mechanisms are influenced primarily by changes in floral resource availability—including the abundance, diversity, and nutritional composition of floral rewards—which in turn affect foraging behavior, reproductive outcomes, and shape population trajectories (Vaudo et al. 2024; Domínguez‐Garcia et al. 2024). While these dynamics have been extensively studied in social bees, especially the Western honey bee ( *Apis mellifera* ), comparatively little attention has been given to solitary bee species, despite their considerable ecological and economic importance.

Solitary bees account for the vast majority of global bee diversity, estimated at 80% of all species (Pitts‐Singer et al. 2018). Unlike some social and semi‐social species, solitary bees do not form colonies, and instead, population persistence relies on individual females to construct and provision nests, making their reproductive success more directly tied to local resource conditions (Pitts‐Singer et al. 2018; Orr et al. 2021). Among solitary bees, *Osmia lignaria* Say (Megachilidae), commonly known as the Blue Orchard Bee (BOB), is one of the most important and agriculturally relevant native pollinators in North America. Its congeneric species ( *O. cornuta* , *O. rufa* , and *O. bicornis* ) are important European pollinators (Magnin et al. 2025; Krunić and Stanisavljević 2013). This species has been increasingly integrated into agricultural systems as an alternative or supplement to pollination by honey bees *A. mellifera* , particularly in the pollination of orchards such as almonds and stone fruits (Bosch and Kemp 2002; Pitts‐Singer et al. 2018; Bosch et al. 2006; McCabe et al. 2024). Its efficiency in crop settings stems from several key traits: early spring emergence, short flight periods that coincide with bloom, high floral constancy, and strong adaptability to artificial nesting substrates (Bohart 1972; Artz et al. 2013; Bosch et al. 2006). It is worth noting that *O. lignaria* 's pollination performance equals or exceeds that of honey bees in raspberry crops and in strawberries—producing bigger berries and at a faster growth rate (Horth and Campbell 2018). It also surpasses other *Osmia* species for canola and rapeseed systems (Abel et al. 2003). Furthermore, *O. lignaria* can forage under cool weather conditions, which are suboptimal for honey bee performance (Bosch and Kemp 2002).

Over the past several decades, research on the developmental, behavioral, and ecological characteristics of *O. lignaria* has expanded considerably, yet much of this work remains scattered across studies focused on specific traits and particular contexts. These studies show that *O. lignaria* is highly sensitive to environmental conditions—its developmental timing and survival are strongly influenced by temperature (Bosch and Kemp 2002; Kemp and Bosch 2005) while nesting success in artificial blocks is influenced by nest architecture and placement (Artz et al. 2013; Tepedino and Torchio 1994; Phillips and Klostermeyer 1978). Moreover, foraging patterns exhibit strong but flexible preferences, often prioritizing nutritionally rich pollen sources over floral abundance or proximity (Kraemer and Favi 2005; Sheffield, Westby, Kevan, and Smith 2008; Williams and Tepedino 2003). Like all other pollinator species, *O. lignaria* also confronts multiple stressors, from pesticides and pathogens (Youssef and McManus 2001; Peterson et al. 2021; KA et al. 2023) to landscape‐level changes that affect resource continuity and diversity (Boyle et al. 2020).

Despite increasing interest in *O. lignaria* in managed systems, its biology, foraging ecology, and responses to environmental variability have not yet been synthesized in the literature. Existing reviews typically focus either on general solitary bee biology or on specific management applications in agricultural systems (e.g., Bosch and Kemp 2002), limiting our understanding of how widespread the described patterns are. Such integration is both timely and necessary, not only for guiding best practices in managed pollination but also for identifying key research needs for solitary bee conservation by using *O. lignaria* as an emerging model system. Here, we aim to fill this gap by bringing together decades of research on *O. lignaria* , covering its developmental plasticity, nesting behavior, foraging ecology, and responses to environmental and anthropogenic stressors. On October 4, 2023, we performed a search on Web of Science to identify all publications with the phrase “ *Osmia lignaria* ” in the title. This search yielded 95 results, and we reviewed and extracted the relevant information, summarized below. In addition, we incorporated data from a complementary database that compiled searches from both Web of Science and Google Scholar searches. For this second database, searches included papers containing Osmia* or *Osmia lignaria* in the title, key‐words, or body of the paper. We hand‐screened each abstract for relevance to *Osmia lignaria* 's ecology, behavior, or life history. This resulted in a total of 561 papers, of which 349 were deemed relevant. These combined sources formed the basis for our comprehensive review. Our goal is for our synthesis to serve as a comprehensive resource for researchers, growers, and land managers interested in studying and conserving this and other ecologically important solitary native pollinators.

### 1.1 Temperature‐Mediated Developmental Plasticity

**FIGURE 1:**
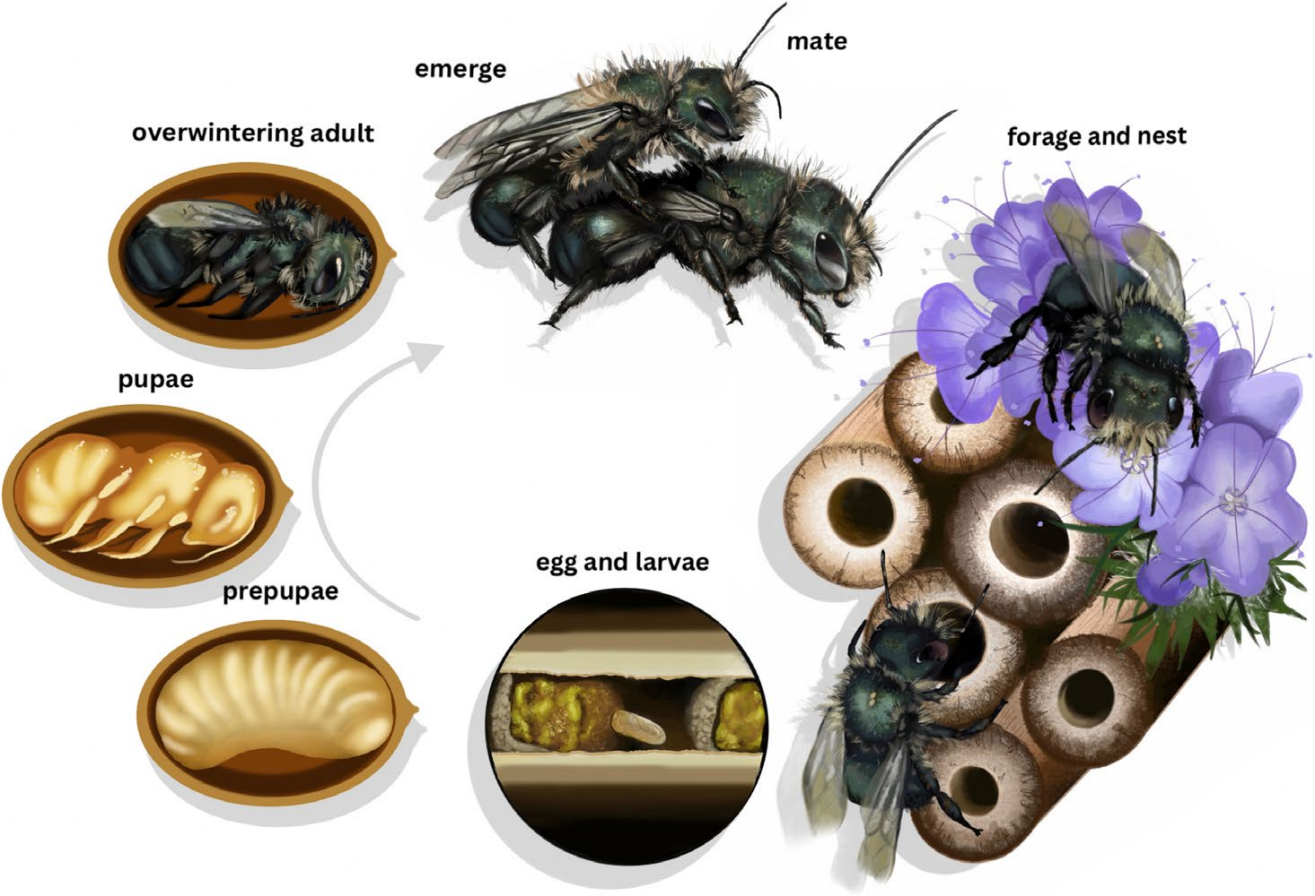
*Osmia lignaria* foraging on a flower of *Phacelia tanacetifolia* to illustrate the life cycle of an *Osmia* bee, with adults emerging in early spring, followed by nesting and pollen provisioning, larval development in summer, and overwintering before the next emergence. Original illustration by Natalie Van Pelt.

*Osmia lignaria* typically emerges in spring, from March to June (Bosch and Kemp 2002), coinciding with the blooming period of many plants in natural ecosystems. Unlike some social bees, *O. lignaria* completes its entire life cycle within a single year, overwintering in a diapause state as a fully developed adult within its cocoon (Figure 1). Favorable temperatures trigger emergence in early spring (Bosch and Kemp 2002), with adults active only for several weeks to mate, forage, and provision nests for the next generation. One of the most valuable traits of *O. lignaria* for agricultural use is its developmental plasticity, which human management can control through temperature regulation (Bosch et al. 2000). In an agricultural setting, by carefully managing overwintering and spring incubation conditions, growers can accelerate emergence and align it with crop blooming, such as in almonds, which begin blooming as early as February. As a result, *O. lignaria* is established as an economically important pollinator species to almonds and other crops that flower in early spring. However, there are trade‐offs between temperature and survival rates, with low post‐cocoon developmental temperatures (18°C) leading to lower survival rates and higher temperatures (26°C–29°C) supporting higher survival rates (Kemp and Bosch 2005). Controlled warming not only benefited survival rates but also increased developmental speed, with bees reared at warmer developmental temperatures (29°C) completing development in approximately 60 days—twice as fast as those reared at 18°C (Bosch and Kemp 2000). When constant temperatures are not feasible, fluctuating conditions averaging 22°C may also accelerate development, primarily by shortening the prepupal dormancy phase (Bosch and Kemp 2000). Accelerating development through either mechanism allows bees to be wintered earlier, enabling them to complete cold dormancy in time to emerge and pollinate early‐blooming crops such as almonds (Bosch and Kemp 2000). Furthermore, warmer pre‐wintering temperatures significantly shortened development without reducing survival of *O. lignaria*, with bees wintered at 7°C emerging earlier than those kept at 4°C. However, prolonged exposure to warm winter conditions, especially above 7°C for more than 150 days, can negatively affect adult performance by causing premature emergence and the depletion of fat body storage (Bosch and Kemp 2003; Bosch et al. 2010).

The mechanisms for the relationship between development and wintering temperatures have been explored in comparative studies of wintering physiology where *O. lignaria* gradually increaseas oxygen consumption and showed continuous weight loss throughout winter, unlike the prepupal *Megachile rotundata* , which maintained stable, low metabolic rates (Kemp et al. 2004). These distinct overwintering strategies demonstrate how metabolic regulation during dormancy influences energy use and survival rates at emergence (Kemp et al. 2004). Additionally, outdoor wintering in sheltered areas protected from extreme temperatures has been shown to improve survival, especially for locally reared bees, making it a cost‐effective method that synchronizes emergence with bloom while avoiding costly cold storage (Sheffield, Westby, Kevan, and Smith 2008). Moreover, controlling for heat accumulation in the spring can help bees emerge more in sync with bloom, even in regions with cooler springs, where heated field incubation boxes have been effectively utilized to advance bee emergence without increasing mortality (Pitts‐Singer et al. 2008; McCabe et al. 2024). These findings highlight *O. lignaria* 's remarkable developmental plasticity, making it a highly suitable native pollinator for early blooming crops and those with short bloom periods (Bosch et al. 2000; Bosch and Kemp 2000, 2003). However, this plasticity may vary among geographic regions, reflecting populations' adaptability to their local environments.

*Osmia lignaria* currently comprises two recognized subspecies: *O. lignaria* Say and *O. lignaria propinqua* Cresson. The first one occurs primarily throughout eastern North America and parts of Canada, where this subspecies has also been investigated for orchard pollination and management applications in eastern fruit production systems (Kraemer et al. 2014). However, most research and management efforts have focused on *O. lignaria propinqua*, which occurs throughout the western United States. *O. lignaria propinqua* populations span diverse ecoregions throughout the region, including California, Utah, Washington, Idaho, Oregon, Arizona, and Montana. However, most managed propagation occurs in Washington and Utah. Genetic analyses have revealed low but significant differentiation between populations from Utah and Idaho, suggesting limited interbreeding and regional adaptations (Koch et al. 2023). Physiological studies further support this, showing that bees from California, Utah, and Washington exhibit distinct developmental responses when reared under identical thermal conditions, likely reflecting adaptations to their native climates (Pitts‐Singer et al. 2014; Scalici et al. 2023). For instance, California bees developed more slowly but had higher survival rates under California‐like conditions compared to bees from Utah and Washington, which developed faster but suffered higher mortality (Pitts‐Singer et al. 2014). Thus, *O. lignaria propinqua* populations from different geographic regions exhibit distinct developmental responses to thermal conditions, underscoring the necessity for region‐specific management strategies to optimize pollination efficiency (Scalici et al. 2023).

### 1.2 Nesting Behavior

Under natural conditions, *Osmia lignaria* females seek abandoned beetle burrows, dead logs, hollow reeds and stems, stumps, or even unattended active nests of other conspecific females to provision brood cells for their offspring (Tepedino and Torchio 1994; Bosch and Kemp 2002). Each female provisions an average of five brood cells per nest (Phillips and Klostermeyer 1978), and typically completes two to four nests during their lifetime. After selecting a nesting location, the female starts collecting mud and soil to construct nest partitions that will separate each brood cell (Rust 1993). Research on soil texture preferences shows that *O. lignaria* females use a wide range of soil types (e.g., loam, sandy loam, and clay), often incorporating different textures within the same partition (Pinilla‐Gallego et al. 2018). Ideally, each brood cell contains an egg laid on top of a pollen provision, yet some partitions may occasionally be left without eggs, which are considered unsuccessful provisions. Upon hatching from the egg chorion, larvae will consume the pollen supply and progress through a prepupal and pupal stage before overwintering within the cocoon as fully metamorphosed adults (Helm et al. 2017). In addition to gravity, cocoon orientation may be influenced by several factors, including tunnel diameter, larval size, pollen placement, and position within the tunnel. Misdirected cocoons are more common in horizontal nests and are often associated with smaller, male‐producing cells, though adult bees can usually still emerge successfully regardless of the cocoon orientation within nests (Torchio 1980).

Because *O. lignaria* readily accepts pre‐existing wood cavities, growers have used wooden blocks with drilled holes since the 1950s to attract and manage these bees for crop pollination (Bohart 1972; Levin 1957). While alternative materials such as milk cartons and styrofoam have been tested, *O. lignaria* consistently prefers wood blocks and reeds, particularly when placed in sun‐exposed (northeast‐facing) shelters that likely enhance morning foraging activity (Torchio 1982a; Pinilla‐Gallego et al. 2022). Wood nests also yielded a significantly more balanced sex ratio (closer to 2:1 males to females) than milk cartons, which were more male‐biased (Torchio 1982a). However, cavity size (straw diameter) seems to strongly influence sex ratio, with wide‐diameter holes producing more females (the larger sex) than narrow‐diameter holes (Rust 1998; Tepedino and Torchio 1989). This may reflect the greater space available in wider cavities for larvae to consume larger provision masses before feeding ends, since metamorphosis in *O. lignaria* is cued by food absence rather than a critical minimum weight (Helm et al. 2017). The density and design of artificial wooden nest boxes also affect *O. lignaria* 's nest occupancy and reproductive success highlight them as important factors to consider in managed systems. In almond orchards, higher retention and reproductive rates were observed when using more boxes with fewer cavities each (e.g., 100 cavities) compared to fewer boxes with many cavities (e.g., 400 cavities) (Artz et al. 2013). This pattern may reflect improved visibility, reduced crowding, and greater ease of nest relocation after foraging (Artz et al. 2014). In addition, bee recovery rates improved markedly—from 30% to 80%—after the addition of supplemental water to nearby soil patches, which likely facilitated mud collection for nest construction (Boyle et al. 2020).

Besides cavity density, the spatial placement of nest boxes also influences the reproductive success of *O. lignaria* , with bees nesting in centrally clustered boxes exhibiting lower propagation rates than those in more evenly distributed or peripheral boxes (Boyle and Pitts‐Singer 2017). Thus, the organization and location of nest boxes in orchard landscapes are important to support populations of *O. lignaria* that can sustainably expand over time and produce more female offspring than originally released (Bosch et al. 2006). Transportability remains a significant practical limitation for *O. lignaria* relative to honey bees, as females frequently abandon nests and fail to reestablish following relocation (Torchio 1991). Although nest shelters mounted on flatbed trailers have shown some promise in mitigating these losses (Torchio 1991), developing reliable translocation methods alongside improving cocoon availability for commercial deployment remain critical priorities to expand the use of *O. lignaria* as an alternative pollinator.

Beyond external structure, internal nest materials also affect reproductive success. Although cardboard tubes are commonly inserted into drilled holes to facilitate cocoon removal, *O. lignaria* females preferred natural reed substrates in orchard trials, likely due to cues of prior use from the previous generations (Pinilla‐Gallego et al. 2022). Reusing wood blocks from natal nests increased female site fidelity and resulted in a 240% population increase in a single generation (Torchio 1982b), which suggests a role for chemical cues in nest site recognition. For example, *O. lignaria* females mark nest entrances with hydrocarbons and wax esters, enabling individual recognition within crowded aggregations (Guédot et al. 2006). These bees are significantly attracted to the female cocoon (Pitts‐Singer 2007), suggesting that species‐specific or female‐associated chemical signals (e.g., secretion of Dufour's gland) may influence nesting site fidelity (Stanley‐Stahr and Pitts‐Singer 2023; Buckner et al. 2009; Pitts‐Singer et al. 2012) rather than relying only on visual signals (Fauria and Campan 1998). The establishment success of *O. lignaria propinqua* in orchards has been relatively high, likely due to its tendency toward more localized nesting behavior compared to more widely dispersing species (Torchio and Asensio 1985). These findings highlight the importance of optimizing nest materials, density, chemical cues, and release strategies to improve the retention and reproductive success of *O. lignaria* , and other *Osmia* species, in managed systems. Aiming to optimize managed populations for larger body size—given its association with increased fecundity and foraging efficiency—heritability studies in greenhouse environments have shown that body size in *O. lignaria propinqua* is moderately heritable in males (0.34) but lower in females (0.15), suggesting limited potential for selective breeding to enhance size, especially in female bees (Tepedino and Torchio 1994). This low heritability in females may reflect the outsized influence of environmental factors on offspring body size, particularly maternal provisioning behavior, since larval growth in *O. lignaria* is tightly coupled to the quantity of provisions supplied rather than to a genetically determined size threshold (Tepedino and Torchio 1994).

Standardized wooden blocks filled with paper straws are commonly used by scientists as trap nests for *O. lignaria* , attracting free‐living females to nest and enabling easy removal of pollen provisions and offspring for further analysis. This setup has facilitated numerous studies on nesting behavior, habitat preferences, and species interactions. For instance, trap nests deployed across eight habitat types in California's Sierra Nevada foothills revealed that *O. lignaria* nested most successfully in oak woodland and chaparral at moderate elevations (200–700 m), where water and floral resources, especially lupines (*Lupinus* spp.), were abundant (Guisse and Miller 2011). As ecological monitoring tools, trap nests have also documented diverse communities of cavity‐nesting insects, including other bee species such as *Hoplitis albifrons* , wasps like *Euodynerus* spp. or *Monobia quadridens* (Byers 1972), and a range of natural enemies, such as the predatory beetles *Trichodes ornatus* , the parasitic fly *Physocephala* sp., and the cleptoparasitic bee *Stelis montana* (Guisse and Miller 2011). Both eastern and western subspecies of *O. lignaria* have also been observed using these nests, underscoring the utility of this method for monitoring natural populations (Guisse and Miller 2011). Trap‐nesting studies have also helped understand how resource availability influences reproductive outcomes and parasitism. For example, there is a potential trade‐off between cavity availability and nest retention, whereby very low cavity numbers (fewer than 96 cavities) were associated with elevated parasitism rates by the cuckoo bee *S. montana* and the beetle *Tricrania stansburyi* (Farzan 2018), whereas cavity numbers exceeding 100 cavities appeared to reduce bee retention (Artz et al. 2013). These results highlight how nesting resource density influences vulnerability to natural enemies in *O. lignaria* populations inhabiting natural ecosystems.

Unlike social bees, each *O. lignaria* female independently completes all nesting tasks. Sex is determined by the mother, who can choose whether or not to fertilize an egg. Fertilized eggs develop into diploid females, while unfertilized eggs become haploid males, a system known as haplodiploidy. In natural populations, sex ratios are typically male‐biased, but females receive larger pollen–nectar provisions and are placed deeper within the nest, reflecting a greater maternal investment in daughters (Williams et al. 2025; Levin 1966). However, the reproductive output in *O. lignaria propinqua* is shaped by seasonal and aging dynamics, as shown by early natural history experiments. For instance, *O. lignaria propinqua* produces more offspring of both sexes early in the nesting season, with female offspring concentrated at the beginning. Later in the season, parental investment shifted toward males, and offspring size declined over time, with a more pronounced decrease in females (Tepedino and Torchio 1982). While the species can successfully reproduce and survive using a single floral resource such as meadowfoam ( *Limnanthes alba* ), survival and female offspring production can decline at higher bee densities due to resource limitations (Jahns and Jolliff 1991b). Although total nest output may increase with higher density, female production per individual decreases and sex ratios shift toward males, likely due to resource scarcity (Jahns and Jolliff 1991b). Consistent with these patterns, *O. lignaria propinqua* shifts sex allocation seasonally, producing more females early in the season and smaller males later as floral abundance declines (Torchio and Tepedino 1980). These density‐ and season‐dependent reproductive strategies underscore the importance of effectively managing both population size and floral resources to optimize pollination services in agricultural settings. Moreover, provisioning rates appear strongly tied to resource abundance, with females provisioning the most in mid‐season, when floral resources are at their peak (Williams and Tepedino 2003).

### 1.3 Foraging and Pollination

*Osmia lignaria has* been shown to be an effective pollinator across several crop systems, including sweet cherries (Bosch et al. 2006), pears (McCabe et al. 2023), almonds (Torchio 1981; Pitts‐Singer et al. 2018), berries (Horth and Campbell 2018), apples (Sheffield 2014), and various herbaceous crops (Abel et al. 2003; Jahns and Jolliff 1991a). Moreover, the availability of alternative floral resources after crop bloom and exposure to avian predators can influence *O. lignaria* 's foraging success (Bosch et al. 2006). For instance, in certain crops, the pollinating efficiency of *O. lignaria* is comparable to that of honey bees ( *Apis mellifera* ) (Andrikopoulos and Cane 2018), whereas *O. lignaria* has been shown to outperform honey bees both in sweet cherry (Bosch et al. 2006) and in almond orchards (Pitts‐Singer et al. 2018), particularly under cold weather conditions that limit honey bee activity. The presence of *O. lignaria* also increased the size and growth rate of herbaceous crops such as strawberries (Horth and Campbell 2018). Finally, *Osmia lignaria* has also been found to be a highly effective pollinator of *Brassicaceae* crops under caged conditions (Abel et al. 2003).

It is important to recognize the context dependence of *O. lignaria* 's pollination effectiveness, as its contribution may be limited in systems where baseline pollination from honey bees is already sufficient. For example, supplementing tart cherry orchards with managed *O. lignaria* did not significantly increase fruit set or yield relative to orchards pollinated solely by honey bees (Boyle and Pitts‐Singer 2019). These results suggest that adding solitary bees in systems where honey bee pollination is already near saturation may offer limited additional benefits. In commercial blueberry fields, *O. lignaria* collected little blueberry pollen, instead favoring alternative sources such as black cherry ( *Prunus serotina* ) and clovers (*Trifolium* spp.), indicating it may be a poor candidate for targeted blueberry pollination (Pinilla‐Gallego and Isaacs 2018). Similarly, when nesting within apple orchards ( *Malus domestica* ), *O. lignaria* prioritized redbud ( *Cercis canadensis* ) pollen when available, highlighting the influence of the composition of the surrounding landscape on the foraging outcomes (Kraemer et al. 2014; Rust 1990). Thus, orchard managers considering *O. lignaria* must account for the floral composition of surrounding habitats, as the presence of certain preferred plants like redbud could significantly impact pollinator behavior and effectiveness (Kraemer et al. 2014).

As a bee species that collects pollen from a wide variety of flowering plant species (polylectic), *O. lignaria* commonly forages on several non‐crop floral resources, such as *Salix* spp. (Salicaceae), *Trifolium* spp. (Fabaceae), and *Prunus* spp. (Rosaceae) (Pitts‐Singer et al. 2018). It also exhibits experimentally demonstrated preferences for non‐crop plants such as *Phacelia tanacetifolia* (Boraginaceae) and *Collinsia heterophylla* (Plantaginaceae), which can successfully support offspring provisioning (Argueta‐Guzmán et al. 2026). Endorsing floral selectivity, *O. lignaria* has been shown to travel longer distances to access nutritionally superior resources, rather than foraging solely based on proximity or floral density (Williams and Tepedino 2003). Consequently, management strategies developed for *O. lignaria* , including the incorporation of wildflower strips and post‐bloom forage resources, may also provide habitat for other solitary bee species.

Several studies further demonstrate the importance of alternative floral resources for sustaining *O. lignaria* populations once crops have finished blooming. For instance, following apple bloom (*Malus* spp.) in Nova Scotia, *O. lignaria* can maintain its reproductive output by foraging on bigleaf lupine ( *Lupinus polyphyllus* ), which supports continued provisioning for offspring (Sheffield, Westby, Smith, and Kevan 2008). Thus, over multiple seasons, bees initially collected mostly apple pollen, but shifted almost entirely to lupine once apple bloom ended (Boyle et al. 2020; Trostle et al. 2025). As a result, nests placed near lupine plots exhibited significantly higher reproductive success, with some recovery rates exceeding 200%, highlighting the value of integrating post‐bloom floral resources into orchard landscapes to promote pollinator persistence and productivity (Sheffield, Westby, Smith, and Kevan 2008). A similar positive influence of alternative floral resources on *O. lignaria* populations has been observed in almond orchards, while *O. lignaria* frequently visited wildflower strips placed adjacent to almond plots during and after almond bloom, and significantly more nests and viable progeny were recovered at orchard edges closest to the wildflowers (Boyle et al. 2020). Pollen analysis revealed that after almond bloom, bees collected up to 72% of their pollen from *Phacelia* species, underscoring the importance of alternative floral resources for sustaining reproductive activity (Boyle et al. 2020). Thus, the effectiveness of *O. lignaria* as a crop pollinator depends not only on its performance during crop bloom but also on the availability of supportive floral and habitat resources beyond the crop's flowering period.

Finally, evidence is starting to accumulate showing that *Osmia* foraging behavior is shaped by olfactory learning and discrimination (Howell and Alarcón 2007), potentially influenced by floral volatiles derived from microbial activity (Martin et al. 2022), as seen to affect other generalist bees (Rering et al. 2018). As *O. lignaria* interacts with flowers during foraging, these encounters facilitate the transmission of microbes that contribute to shaping its microbiome (McFrederick et al. 2012, 2017; Adler et al. 2021; Argueta‐Guzmán et al. 2025). Furthermore, the foraging behavior of *O. lignaria* is also strongly influenced by floral resource availability and nutritional quality (Rust 1990; Kraemer and Favi 2005). In an experimental setting, *O. lignaria* was superior in choosing feeders associated with nectar‐like rewarded odors when compared to the highly commercial solitary bee *Megachile rotundata* (Stanley‐Stahr and Pitts‐Singer 2023).

### 1.4 Stressors

Like all organisms, *Osmia lignaria* is exposed to multiple environmental stressors that affect individuals' health, performance, and the persistence of their populations (Klein et al. 2017). Thus far, as with most other pollinator species, the major stressors studied for *O. lignaria* are pathogens, pesticides, and heat stress. Among the pathogens affecting *O. lignaria* and other megachilid bees, chalkbrood disease, caused by fungal species in the genus *Ascosphaera*, has gained recent attention due to its potential spillover to *O. lignaria* from honey bees (KA et al. 2023). *Ascosphaera* species are exclusively associated with bee nests, with interactions ranging from neutral to negative (Anderson et al. 1998). In honey bees, *Ascosphaera apis* causes chalkbrood disease, while *A. aggregata* is known to infect the alfalfa leaf‐cutting bee *Megachile rotundata* . Infection typically begins with the ingestion of ascospores, which germinate in the larval midgut. The fungus then breaches the gut epithelium, spreads into the hemocoel, and leads to systemic mycosis. There is evidence suggesting potential cross‐infectivity of *Ascosphaera* species to *O. lignaria* (Youssef et al. 1985), and recent studies have detected *Ascosphaera* in both the larvae and pollen provisions (KA et al. 2023; Crowley and Schaeffer 2024). Notably, non‐native chalkbrood fungi have been observed in native mason bee nests in North America, with incidence increasing in areas where non‐native mason bees are more abundant. Agricultural landscapes may amplify fungal spillover, increasing the risk to native mason bees (KA et al. 2023). Further experimental evidence suggests that *O. lignaria* is susceptible to chalkbrood under controlled conditions—eggs and fifth instar larvae inoculated with *Ascosphaera* spores and reared at lower temperatures (21°C) showed high incidence of chalkbrood (Torchio 1992).

Bee exposure to harmful chemicals stems from industrialized agricultural practices aimed at pest control (e.g., the application of neonicotinoids and pyrethroids), as well as from airborne particulate matter originating in livestock operations, such as macrocyclic lactones (e.g., abamectin and ivermectin, widely used as antiparasitic drugs) (Green et al. 2023; Peterson et al. 2021). Among these, foliar pesticides and insecticides of the neonicotinoid class exhibit 47‐fold higher toxicity than other classes, posing the greatest risk to all bees, including *O. lignaria* (Scott‐Dupree et al. 2009). Experimental evidence indicates that neonicotinoids are approximately 40% more toxic to *O. lignaria* than to honey bees (Peterson et al. 2021), and have been shown to delay larval development (Abbott et al. 2008), increase adult mortality (Green et al. 2023), and reduce nesting success (Fortuin et al. 2021). In addition, pyrethroids and macrocyclic lactones—once assumed to be confined to livestock environments—also exhibit similarly high toxicity (Peterson et al. 2021). Studies show that both foliar and airborne chemical residues of neonicotinoids (thiamethoxam, imidacloprid, and clothianidin) and macrocyclic lactones (abamectin and ivermectin) can deliver lethal doses to *O. lignaria* , highlighting a critical flaw in regulatory risk assessments that rely solely on honey bee data and likely underestimate risks to solitary pollinators (Peterson et al. 2021) Moreover, contaminated nesting materials represent an additional exposure route for solitary bee larvae; laboratory experiments demonstrated that pesticides in leaves and soil can transfer into larval provisions, with chlorpyrifos concentrations in alfalfa leafcutting bee provisions reaching levels in the range of concern, although transfer from soil into *O. lignaria* provisions was minimal (Luu et al. 2025). Beyond insecticides, studies have also assessed the effects of fungicides, though less frequently. Fungicides are typically applied during flower blooming, which may increase bee exposure to these chemicals (Durant et al. 2021). Although *O. lignaria* often continues to forage normally in the presence of fungicides (Ladurner et al. 2008), this behavior should not be mistaken for immunity, as exposure to fungicides such as Rovral 4F, Pristine, and N‐90 led to impaired female nest recognition abilities (Artz and Pitts‐Singer 2015). Moreover, even when fungicides' effects on foraging behavior or mortality are absent, delayed impacts on survival have been documented, particularly with compounds such as propiconazole and captan (Ladurner et al. 2005).

Beyond individual stressor effects, the combined impacts of pesticide exposure and resource limitation on *O. lignaria* behavior and offspring production have also been investigated. Insecticide exposure and floral resource limitation act additively to reduce reproduction in *O. lignaria* , with each stressor independently impairing nesting and offspring production (Stuligross et al. 2023). At the behavioral level, resource‐limited bees made fewer but longer foraging trips and misidentified their nests more frequently, while insecticide‐exposed bees showed a general reduction in foraging activity, effects that together point toward compounding reductions in pollination services and population persistence (Stuligross et al. 2023). Furthermore, adult exposure to the neonicotinoid imidacloprid suppressed nectar and pollen feeding in *O. lignaria* , subsequently impairing ovary development and oocyte maturation, with carryover effects from larval exposure further altering adult feeding behavior (Stuligross 2026).

Finally, rising temperatures and increasingly frequent heatwaves are among the most detrimental effects of climate change, acting as additional stressors for *Osmia lignaria* and other bees. Solitary bees lack the nest thermo‐regulating mechanisms of their social counterparts, leaving their developing offspring fully exposed to ambient thermal conditions and making early life stages particularly vulnerable (Melone et al. 2024). Recent experimental work demonstrates that even short periods of extreme heat can be lethal; heatwaves reaching 37°C increased larval mortality by more than 130% and caused marked delays in development, while even moderate heatwaves shifted mortality earlier in development (Melone et al. 2024). These delays can extend larval exposure to other stressors later in the season and reduce the likelihood of successful emergence (Bosch and Kemp 2003; Bosch et al. 2010). Beyond acute heatwaves, chronic warming also imposes strong sublethal and lethal effects on solitary bees (CaraDonna et al. 2018). Field warming experiments show that increased temperatures delay adult emergence, increase phenological variance, reduce body mass and fat content, and dramatically increase mortality (30%–75%) in related *Osmia* species (CaraDonna et al. 2018). Such shifts are likely to disrupt synchrony with floral resources, impair mating opportunities, and reduce population persistence under future climate conditions (CaraDonna et al. 2018). These results highlight the need for integrated studies that simultaneously examine multiple stressors across groups beyond social bees and examine how different environmental conditions may amplify their impacts on solitary bee health, population persistence, and pollination services.

## 2 Future Directions

Beyond its role as a managed orchard pollinator, *O. lignaria* has several traits that make it a valuable model system in ecology. Its discrete nesting biology, measurable reproductive investment at the individual level, and amenability to experimental manipulation in both laboratory and field settings make *O. lignaria* particularly well‐suited for ecological research across ecological scales (Salerno et al. 2026; Crall and Gaiarsa 2026). Future work should focus on elucidating how multiple stressors interact with floral resource availability to influence survival and population persistence in wild populations, as well as how wild and managed populations may acclimate to future environmental conditions. This work could leverage the recently sequenced *O. lignaria* genome (USDA‐ARS 2022; NCBI 2026) to explore the role of genetic variation among populations in response to environmental stressors (e.g., pathogens, pesticides, rising temperatures), potentially leading to the identification of genes associated with tolerance and recovery to these variables. As genomic resources for solitary bees continue to expand (USDA‐ARS 2022), comparative genomic approaches may help identify conserved stress‐response pathways shared across bee taxa. Combined, these developments position *O. lignaria* as a valuable system for integrating genomics with experimentally tractable studies of environmental responses relevant across solitary bee species. Furthermore, because individual females independently construct and provision nests in a sequential and spatially organized manner, ecologists can directly quantify temporal patterns of resource allocation (e.g., within‐season variation), provisioning effort, and reproductive success to test predictions from optimal foraging theory at the individual and population levels. In turn, understanding how resource availability and floral abundance influence reproductive outcomes could help improve cocoon production in agricultural settings. *O. lignaria* is commercially distributed and deployed as overwintering cocoons, and insufficient supply from distributors coupled with limited transportability likely contributes to the continued dominance of honey bees in commercial pollination. Together, these future directions position *O. lignaria* as a valuable system for bridging applied pollinator biology with broader ecological theory relevant across pollinator taxa.

## Author Contributions

**Natalie Van Pelt:** conceptualization (equal), data curation (equal), investigation (equal), methodology (equal), project administration (equal), visualization (lead), writing – original draft (equal), writing – review and editing (equal). **Magda Argueta‐Guzmán:** writing – original draft (equal), writing – review and editing (equal). **Trizthan Jimenez Delgado:** data curation (equal), investigation (equal), writing – review and editing (supporting). **Lindsie McCabe:** data curation (equal), investigation (equal), writing – review and editing (equal). **Marilia Palumbo Gaiarsa:** conceptualization (equal), data curation (equal), funding acquisition (lead), investigation (equal), methodology (lead), project administration (equal), resources (lead), supervision (lead), writing – original draft (equal), writing – review and editing (equal).

## Funding

M.A.‐G. and M.P.G. thank the support of the California Natural Resources Agency, and M.P.G. acknowledges the support of the National Science Foundation under Grant No. 2437073. This research was supported by the U.S. Department of Agriculture, Agricultural Research Service project plan 2080‐30500‐001‐000D. Mention of trade names or commercial products in this publication is solely for the purpose of providing specific information and does not imply recommendation or endorsement by the U.S. Department of Agriculture.

## Conflicts of Interest

The authors declare no conflicts of interest.

## Data Availability

We did not generate or analyze any new data in this study. All information used in this review is derived from previously published studies, which are cited within the article.
